# Comparison of the antioxidant activities of nonfumigated and sulphur-fumigated *Chrysanthemum morifolium* cv. Hang-ju induced by oxidative stress

**DOI:** 10.1080/13880209.2020.1865409

**Published:** 2021-01-05

**Authors:** Hongyan Ma, Shanshan Liu, Wenshan Qu, Qi Huang, Linyuan Li, Fujiang Chu, Yuyun Zhu, Xinlin Lv, Zhimin Wang, Jingjing Zhu

**Affiliations:** a Key Laboratory of State Administration of Traditional Chinese Medicine for Production & Development of Cantonese Medicinal Materials/School of Traditional Chinese Medicine, Guangdong Pharmaceutical University, Guangzhou, China; b Beijing Center for Physical and Chemical Analysis, Beijing, China; c Guangdong Provincial Key Laboratory of Pharmaceutical Bioactive Substances/School of Life Science and Biopharmaceutics, Guangdong Pharmaceutical University, Guangzhou, China; d National Engineering Laboratory for Quality Control Technology of Chinese Herbal Medicine/Institute of Chinese Materia Medica, China Academy of Chinese Medical Sciences, Beijing, China

**Keywords:** Compositae, drying process, hyperlipidaemia, HUVECs, apoptosis

## Abstract

**Context:**

The traditional drying method, sun drying, for *Chrysanthemum morifolium* Ramat. cv. Hang-ju (Compositae) (HJ) is widely replaced by sulphur fumigation (SF), which has an unknown effect on its efficacy.

**Objective:**

To investigate protective effects of nonfumigated HJ (NHJ) and sulphur-fumigated HJ (SHJ) water extracts against oxidative stress and lipid peroxidation.

**Materials and methods:**

Sprague-Dawley rats were administered high-fat diet to induce hyperlipidaemia and randomly divided into eight groups (*n* = 6): control, fenofibrate, NHJ and SHJ extracts (1, 2 or 4 g crude drugs/kg/d; intragastric administration for 8 weeks). Serum total cholesterol (TC), triglyceride (TG), low-density lipoprotein cholesterol (LDL-C), high-density lipoprotein cholesterol (HDL-C), superoxide dismutase (SOD) and malondialdehyde (MDA) levels were detected. Human umbilical vein endothelial cells (HUVECs) were treated with NHJ and SHJ extracts (50, 100 or 200 μg/mL) for 24 h, followed by oxidized low-density lipoprotein (ox-LDL, 20 μg/mL) for 2 h *in vitro*. Cellular reactive oxygen species (ROS), SOD and MDA levels and apoptosis were evaluated.

**Results:**

NHJ was more effective than SHJ in decreasing serum TG, TC, LDL-C, LDL/HDL and MDA while increasing serum HDL-C and SOD levels at high doses. SHJ (IC_50_=19.9 mg/mL) suppressed HUVEC growth stronger than NHJ (IC_50_=186.7 mg/mL). At 200 μg/mL, NHJ was more effective than SHJ in downregulating ROS and MDA levels, reducing HUVECs apoptosis rate and elevating SOD activity in ox-LDL-treated HUVECs.

**Conclusions:**

SF causes oxidative damage and attenuates antioxidative activity in ox-LDL-treated HUVECs, which promotes lipid peroxidation. SF is not recommended for processing HJ.

## Introduction

The dry capitulum of *Chrysanthemum morifolium* Ramat. (Compositae) is a popular traditional Chinese medicine known as ‘Ju Hua’ in China, that is included in the Chinese Pharmacopoeia as *Chrysanthemi Flos* (Chinese Pharmacopoeia Commission 2015). It is also widely used as an herbal tea, beverage and seasoning due to its unique flavour, colour and health benefits (Lii et al. 2010). Pharmacological studies have shown that *C. morifolium* possesses multiple activities, and its antioxidant activity is of greatest interest. Indeed, several studies have reported that *C. morifolium* extracts have antioxidant activity (Chen et al. 2015; Yuan et al. 2015). Oxidative stress is the common mechanism of many cardiovascular disease (CVD) risk factors, which suggests that it plays a central role in CVD (Madamanchi et al. 2005).

In vascular intimal injury, oxidative stress is likely to be an early transient event that parallels the inflammatory and proliferative phases of the vascular response (Azevedo et al. 2000). A previous study showed that an extract of *Chrysanthemi Flos* can attenuate vascular intimal injury through antioxidative mechanisms (Jin et al. 2009).

Drying is considered the crucial step in postharvest processes due to its importance in limiting enzymatic degradation and microbial growth while preserving the plant’s beneficial properties (Yuan et al. 2015). Traditionally, shade drying, sun drying and hot air drying are the standard preparation methods of *C. morifolium*. However, sulphur fumigation (SF), a fast and cheap method well known for its various benefits, including its ability to shorten drying time; preserve colour; and prevent pests, mould and bacterial contamination, has been commonly employed to replace the natural drying processes of *C. morifolium* in recent years (Kan et al. 2011).

Nevertheless, SF is forbidden because the period needed for SF inevitably has negative effects on the ingredients of herbal drugs, causing alterations in the bioactive components of herbs and consequently affecting their bioactivities and pharmacokinetics (Zhan et al. 2014). In addition, long-term exposure to sulphur dioxide and heavy metals produced by SF can cause an uncomfortable mouth feel and induce respiratory and gastrointestinal symptoms, including cough, chest tightness, throat discomfort and shortness of breath (Duan et al. 2016). Thus, the China Food and Drug Administration has compulsorily demanded that the level of sulphur dioxide residue be lower than 400 mg/kg for 12 TCMs and lower than 150 mg/kg for all other TCMs to ensure their quality, safety and efficacy (Guo et al. 2014). To date, the occurrence of SF-induced chemical alterations in a great deal of TCMs, including *C. morifolium* (Wang S et al. 2014), Astragali Radix (Xing et al. 2018), Radix *Paeoniae alba* (Xu et al. 2018), *Panacis quinquefolii* Radix (Jiang et al. 2020), *Gastrodia* Rhizoma (Kang et al. 2017), *Smilacis glabrae* Rhizoma (Yuan et al. 2019) and *Ginseng* Radix et Rhizoma (Ma et al. 2017), has been verified. Our previous research showed that SF of *C. morifolium* cv. Hang-ju (HJ) causes flavonoid glycosides to transform into aglycons by hydrolysis and results in a significant loss of hydroxycinnamoylquinic acids. However, no investigations have revealed the different effects of nonfumigated HJ (NHJ) and sulphur-fumigated HJ (SHJ) on antioxidation induced by oxidative stress.

Therefore, in the present study, the protective effects of NHJ and SHJ in hyperlipidemic rat model as well as human umbilical vein endothelial cells (HUVECs) exposed to oxidized low-density lipoprotein (ox-LDL) induced oxidative damage were comprehensively investigated. The results provide evidence for the effective and safe application and preparation of *C. morifolium*.

## Materials and methods

Sodium deoxycholate, propylthiouracil and vitellus powder were procured from Sangon Guangzhou Surui Biotechnology., Ltd. (Guangzhou, China), and cholesterol was obtained from Shanghai Lanji Technology Development Co., Ltd. (Shanghai, China). Total cholesterol (TC), triglycerides (TGs), high-density lipoprotein cholesterol (HDL-C), low-density lipoprotein cholesterol (LDL-C), superoxide dismutase (SOD), malondialdehyde (MDA) and reactive oxygen species (ROS) assay kits were purchased from the Nanjing Jiancheng Bioengineering Institute (Nanjing, China).

HUVECs were a kind gift from Professor HL Sun. Ox-LDL was obtained from Sigma (St. Louis, MO). Dimethylsulphoxide (DMSO) and 3-(4,5-dimethyl-2-thiazolyl)-2,5-diphenyl-2-H-tetrazolium bromide (MTT) were purchased from Aladdin (Shanghai, China). Fenofibrate (FEN; 200 mg/tablet) was procured from Laboratories Fournier (Dijon, France). All other chemicals used were of analytical grade.

### Preparation of HJ extracts

Dried NHJ and SHJ were collected by the authors from Bozhou, PR China, in April 2016 and identified by the corresponding author (Jingjing Zhu). Voucher specimens (no. BZHJ201608) were deposited in the Quality Standards Research Center, Institute of Chinese Materia Medica, China Academy of Chinese Medical Sciences, Beijing, China. Dried NHJ and SHJ were extracted with boiling water (2 × 10 L; 3 h each). The aqueous solutions were filtered and vacuum evaporated to dryness. The yield of each sample, i.e., 36.7% and 31.3% for NHJ and SHJ, respectively, was measured by calculating the weight of the dried extract/the weight of primary dried material (w/w %).

### Animals and treatment

In order to exclude the influence of menstruation on blood lipid index (Svanborg and Vikrot 1967), only male Sprague-Dawley rats (190 ± 10 g) were obtained from the Guangdong Medical Laboratory Animal Center (Foshan, China). The animals were housed in a standard laboratory within the Experimental Animal Management Center of Guangdong Pharmaceutical University under controlled temperature (22–25 °C) and humidity (50–60%) and maintained under a 12 h light/dark cycle. All rats were kept in propylene cages and provided *ad libitum* access to standard food and clean water. All experimental procedures were carried out according to the Guidelines for the Care and Use of Laboratory Animals and were approved by the Experimental Animal Management Center of Guangdong Pharmaceutical University.

Animals were kept under observation for a week and randomly divided into two groups. Rats from the two groups were housed in separate cages and were administered different therapies. The blank control (BC) group (*n* = 6) was given normal chow, and the other group (model group, *n* = 48) was administered a high-fat diet (pork oil, 20%; cholesterol, 5%; egg yolk powder, 1%; sodium deoxycholate, 1%; propylthiouracil, 1%; and fructose, 30%) for eight continuous weeks to induce hyperlipidaemia (Chu et al. 2013).

### Cell culture

Primary HUVECs were cultured at 37 °C under 5% CO_2_ for 2–3 days in 75 cm^2^ flasks containing CM-200 medium supplemented with 2% foetal bovine serum, 1 μg/mL hydrocortisone, 10 ng/mL human epidermal growth factor, 3 ng/mL basic fibroblast growth factor and 10 μg/mL heparin. Confluent cells were detached using a trypsin–EDTA solution, and the cells were sedimented by centrifugation at 100 rpm for 5 min at 4 °C. After the supernatant was removed, the cell pellets were resuspended in fresh medium, counted with a haemocytometer, and seeded at a density of 1 × 10^4^ cells per well in a 96-well plate. Using a trypan blue exclusion method, cell viability was determined to be consistently greater than 90% prior seeding of the endothelial cells. The monolayers were used within 2 h after reaching confluence.

### Experimental design and treatments

After the hyperlipidaemia animal model was established, the animals were divided randomly into the following eight groups; a negative control (NC) group (intragastric administration of distilled water), a FEN group (0.02 g/kg), NHJ treatment groups (1, 2 or 4 g crude drug/kg/d) and SHJ treatment groups (1, 2 or 4 g crude drug/kg/d). The rats in the control group were lavaged with pure water and standard food, and the rats in the other seven groups were gavaged with drugs dissolved in water for 8 weeks. The animals were gavaged with a volume of 10 mL/kg once per day. Pentobarbital sodium was used to induce euthanasia after 8 weeks, and 3 mL of blood was collected from the abdominal aorta, kept for 1 h at room temperature and centrifuged for 10 min at 3000 rpm to obtain serum. The concentrations of TC, TG, LDL-C, HDL-C, SOD and MDA were measured strictly according to the kit instructions (Beyotime Institute of Biotechnology, Nantong, China).

Cell growth and the sensitivity of HUVECs to the two HJ extracts were assessed. The HUVECs were preincubated with varying concentrations of NHJ (50, 100 and 200 μg/mL) and SHJ (50, 100 and 200 μg/mL) for 24 h and then treated with ox-LDL (20 μg/mL) for another 2 h. The cells were collected to test for intracellular ROS, MDA and SOD production as well as cell apoptosis (Yu et al. 2010; Chu et al. 2015).

### Equilibrium solubility detection of HJ extracts

In order to make sure that the HJ dose which used *in vitro* experiment does not exceed the equilibrium dose, the equilibrium solubility of the culture medium of NHJ- and SHJ-treated cells was determined by quantifying the concentration of the test sample supernatants (via a centrifugation protocol) and evaluating the values against the calibration curve (Pouton 2006).

### Cell growth and sensitivity study

The cell growth and sensitivity of HUVECs to the two HJ extracts were assessed using the MTT assay (Rojas-Silva et al. 2014). Briefly, HUVECs in suspension were seeded in a 96-well microtiter plate at a density of 1 × 10^4^ cells per well, and grown in a humidified atmosphere of 5% CO_2_ in air at 37 °C. Then, the cells were exposed to varying concentrations of NHJ and SHJ for 24 h. The culture medium was carefully removed and exchanged for fresh medium. MTT solution (5 mg/mL PBS) was then added, and the plate was placed in an optimal atmosphere at 37 °C. The metabolically active cells reduced MTT to blue formazan crystals. After 2 h, the formazan crystals formed were solubilized by incubating cells with DMSO (500 μL). The cell absorbance was read at 570 nm with a microplate reader (BioTek Instruments Inc., Winooski, VT). Cell proliferation was quantified as a percentage of proliferating cells compared to that in the control group (untreated cells), which was set to 100%. Each treatment group was analysed in triplicate wells, and each experiment was performed three times. IC_50_ values (the concentrations of the test compound required to reduce cell survival by 50%) were calculated from concentration–response curves and used as a measure of cellular sensitivity to a given treatment.

### Determination of intracellular ROS production

Approximately, 1.0 × 10^6^/well cells were seeded in a six-well plate overnight. After ox-LDL (20 μg/mL) treatment for 2 h following pre-treatment with varying concentrations of NHJ and SHJ for 24 h, the cells were incubated with CM-DCFH2-DA (10 μM) in the dark at 37 °C for 40 min, washed with PBS and detached with trypsin/EDTA. Cellular fluorescence was analysed by flow cytometry (FCM, Becton Dickinson, Franklin Lakes, NJ). The experiment was performed in triplicate.

### Measurement of intracellular MDA and SOD levels

MDA and SOD levels were assessed to study antioxidation in ox-LDL-induced HUVECs (Zhu et al. 2015). MDA levels were analysed by a commercially available colorimetric assay kit according to the manufacturer’s instructions. Briefly, pre-treated cells were harvested by scraping and homogenized in RIPA buffer on ice. The cell lysates were then centrifuged at 12,000 rpm for 10 min at 4 °C to collect the supernatant. MDA levels were detected at 532 nm using a microplate reader. We used the bicinchoninic acid disodium (BCA) protein assay kit to quantify the protein concentration. MDA content is expressed as nanomoles per milligram of protein.

Intracellular SOD activity was measured with a Total Superoxide Dismutase Assay Kit based on the autooxidation of hydroxylamine. Targeted cells were lysed in PBS by ultrasonic pyrolysis (5 s of sonication plus 5 s of rest; 10 times). Total SOD activity in the homogenates was measured using the hydroxylamine method. The optical density (OD) values of the samples were determined at 550 nm, and each test was repeated at least three times.

### Determination of HUVEC apoptosis by FCM

The necrotic and apoptotic fractions were assessed by annexin V-FITC staining after the induction of cell death using an FCM apoptosis detection kit according to the manufacturer's instructions. HUVECs (2 × 10^5^) were harvested, washed with binding buffer, and resuspended in 100 μL of binding buffer containing annexin-V-FITC (10 μL). After 15 min of incubation at room temperature in the dark, the cells were washed with binding buffer. Just before the samples were analysed by FCM, propidium iodide (PI; final concentration of 5 μg/mL) was added. The cell populations were counted based on the following criteria: annexin V–/PI– cells indicated live cells, annexin V–/PI + cells were necrotic cells, annexin V+/PI– cells represented early apoptotic cells, and annexin V+/PI + cells were late apoptotic cells. The samples were analysed with a FACSCalibur instrument running CellQuest software (Liu et al. 2013).

### Statistical analysis

All data are expressed as the mean values ± standard deviations. For multiple comparisons, one-way analysis of variance (ANOVA) was performed followed by Dunnett's T 3 or Duncan's *t-*tests when appropriate. Differences were considered statistically significant when *p* < 0.05. All statistical analyses were performed with SPSS statistical software (version 13.0 for Windows; SPSS Inc., Chicago, IL).

## Results

### Equilibrium solubility of HJ extracts

The equilibrium solubilities of the culture medium of NHJ- and SHJ-treated HUVECs were 0.462 ± 0.043 and 0.427 ± 0.039 g/mL, respectively, and the difference was statistically significant (*p* < 0.01).

### Effects of HJ extracts on serum lipid, MDA and SOD levels

Serum TG, TC, LDL-C, LDL/HDL levels, MDA content and SOD activity were tested. The levels in the NC group were significantly increased compared to those in the BC group, except the HDL-C level and SOD activity, which were significantly decreased (all *p* < 0.01) (Table 1). These results indicated that the model was successfully established. Compared with the NC group, both the NHJ and SHJ groups exhibited significant decreases in serum TG, TC, LDL-C, LDL/HDL and MDA levels and increases in the serum HDL-C level and SOD activity that were augmented as the NHJ or SJH dose increased (all *p* < 0.01) (Table 1). The results also demonstrated that NHJ was more effective than SHJ in decreasing or increasing these values in hyperlipidemic rats (all *p* < 0.05 or *p* < 0.01). This finding indicates that SF can affect the efficacy of HJ by disturbing serum lipid levels and causing oxidative damage to the body.

**Table 1.** Effects of NHJ and SHJ on lipid regulation and antioxidation in hyperlipidemic rats ($\bar{x}$±*sd*, *n* = 6).

Group	Dose (g/kg)	TG (mmol/L)	TC (mmol/L)	LDL (mmol/L)	HDL (mmol/L)	LDL/HDL	SOD (U/mL)	MDA (nmol/mL)
BC	–	0.71 ± 0.22	2.18 ± 0.25	0.35 ± 0.07	1.28 ± 0.15	0.28 ± 0.04	124.36 ± 10.32	3.71 ± 0.21
NC	–	0.98 ± 0.21^§§^	3.27 ± 0.45^§§^	0.59 ± 0.17^§§^	1.05 ± 0.12^§§^	0.56 ± 0.14^§§^	91.67 ± 2.14^§§^	12.08 ± 0.69^§§^
FEN	0.02	0.69 ± 0.12**	1.94 ± 0.40**	0.37 ± 0.07**	1.22 ± 0.10**	0.30 ± 0.03**	121.08 ± 6.15**	5.74 ± 0.30**
NHJ	1	0.89 ± 0.18**	3.05 ± 0.48^**,##^	0.54 ± 0.09	1.08 ± 0.16^#^	0.53 ± 0.09	94.66 ± 5.13	11.66 ± 1.28
	2	0.82 ± 0.22^**,##^	2.76 ± 0.37^**,##^	0.43 ± 0.09**	1.19 ± 0.09^**,##^	0.36 ± 0.08^**,#^	112.09 ± 6.24**	7.53 ± 0.45^**,##^
	4	0.73 ± 0.13^**,##^	2.24 ± 0.39^**,##^	0.37 ± 0.04^**,##^	1.27 ± 0.11^**,#^	0.29 ± 0.08^**,#^	135.67 ± 7.09^**,##^	4.49 ± 0.68^**,##^
SHJ	1	0.97 ± 0.27	3.29 ± 0.58	0.57 ± 0.10	1.04 ± 0.07	0.52 ± 0.13	90.66 ± 3.21	12.30 ± 1.04
	2	0.91 ± 0.18**	3.04 ± 0.64*	0.50 ± 0.11**	1.12 ± 0.13**	0.45 ± 0.09**	106.15 ± 7.45**	9.55 ± 0.56**
	4	0.85 ± 0.21**	2.98 ± 0.39*	0.45 ± 0.08**	1.16 ± 0.14**	0.38 ± 0.13**	116.32 ± 4.16**	7.60 ± 0.73**

^§§^ *p* < 0.01, indicates significant difference from BC group.

*p < 0.05, **p < 0.01 indicate significant difference from NC group. ^#^*p* < 0.05, ^##^*p* < 0.01 indicate significant difference from SHJ group.

### Sensitivity of HUVECs to HJ extracts

**Figure 1.**
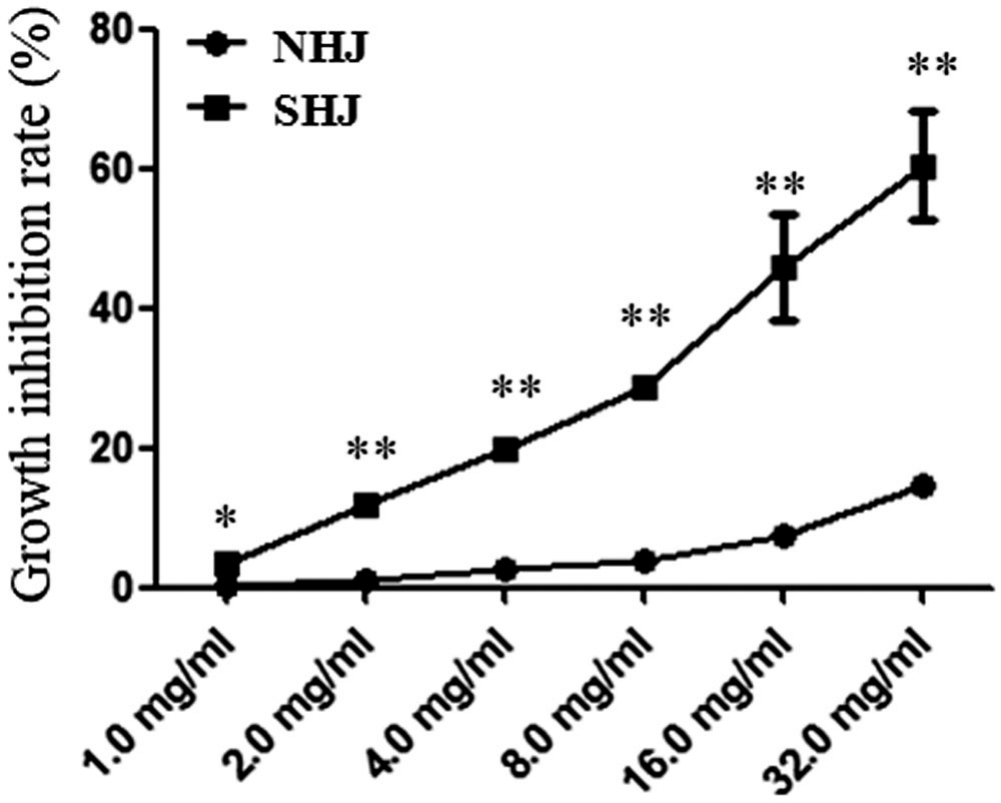
Sensitivity of HUVECs to NHJ and SHJ. Based on the growth inhibition assay, SHJ suppressed the growth of HUVECs more significantly than NHJ. *p < 0.05, **p < 0.01 indicate a significant difference compared to the SHJ group.

The effects of NHJ and SHJ on the proliferation of HUVECs in liquid culture were studied using the MTT assay. HUVECs were treated with varying concentrations of NHJ and SHJ for 24 h. Based on the growth inhibition assay, SHJ suppressed the growth of HUVECs more significantly than NHJ (all *p* < 0.05 or *p* < 0.01) (Figure 1). The IC_50_ values of NHJ and SHJ were calculated to be 186.7 and 19.9 mg/mL, respectively.

### Effects of HJ extracts on intracellular ROS

**Figure 2.**
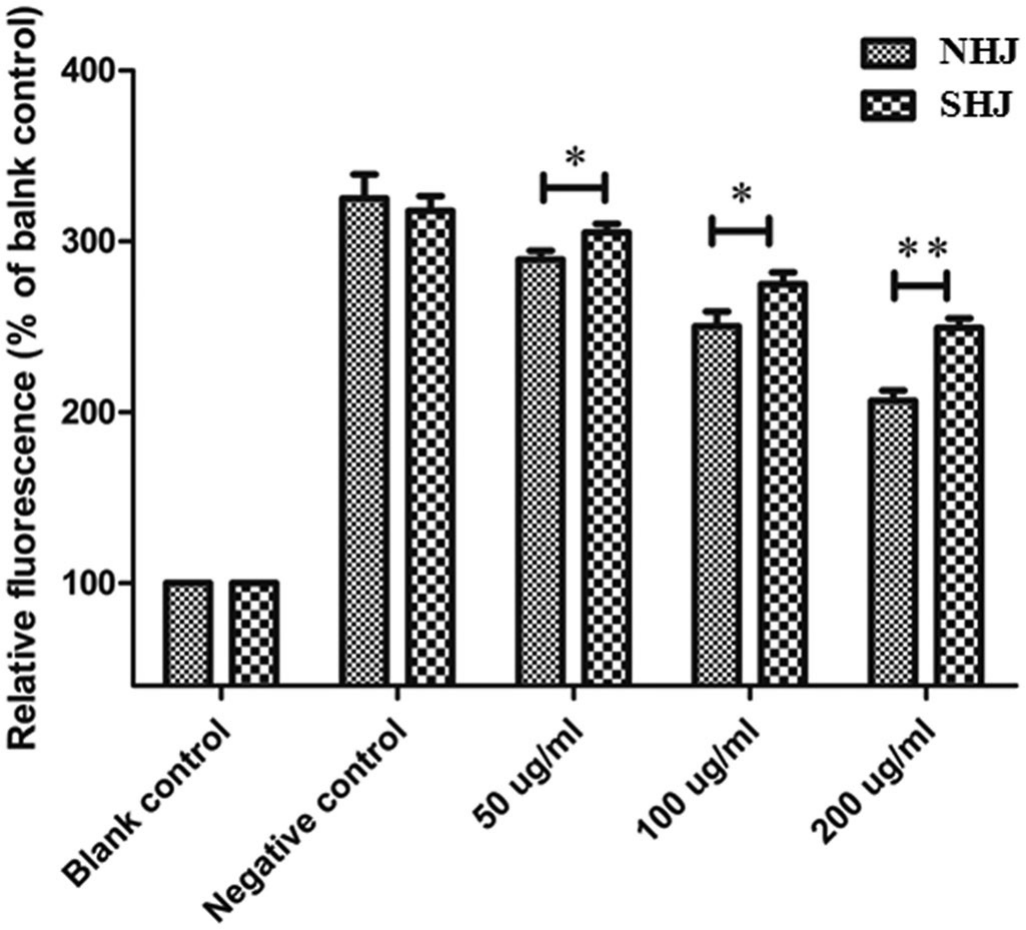
NHJ and SHJ inhibited ROS production in ox-LDL-treated HUVECs. NHJ was more effective than SHJ in decreasing ROS production in HUVECs induced by ox-LDL. *p < 0.05, **p < 0.01 indicate a significant difference compared to the SHJ group.

The average fluorescence intensity of 2′,7′-dichlorofluorescein acetate-stained samples was detected by FCM. Compared with that of the BC group (58.56 ± 5.17), the average fluorescence intensity of the ox-LDL group (188.34 ± 9.06) was significantly enhanced, indicating that ox-LDL treatment increased ROS production in HUVECs. However, compared to ox-LDL treatment alone, pre-treatment with either NHJ or SHJ significantly prevented ox-LDL-induced ROS staining, with the effect increasing as the NHJ or SHJ dose increased (vs. NC group, all *p* < 0.05). The results also showed that NHJ was more effective than SHJ in decreased ROS production, which was induced by ox-LDL in HUVECs (NHJ vs. SHJ, all *p* < 0.05) (Figure 2).

### Effects of HJ extracts on intracellular MDA and SOD activities

**Figure 3.**
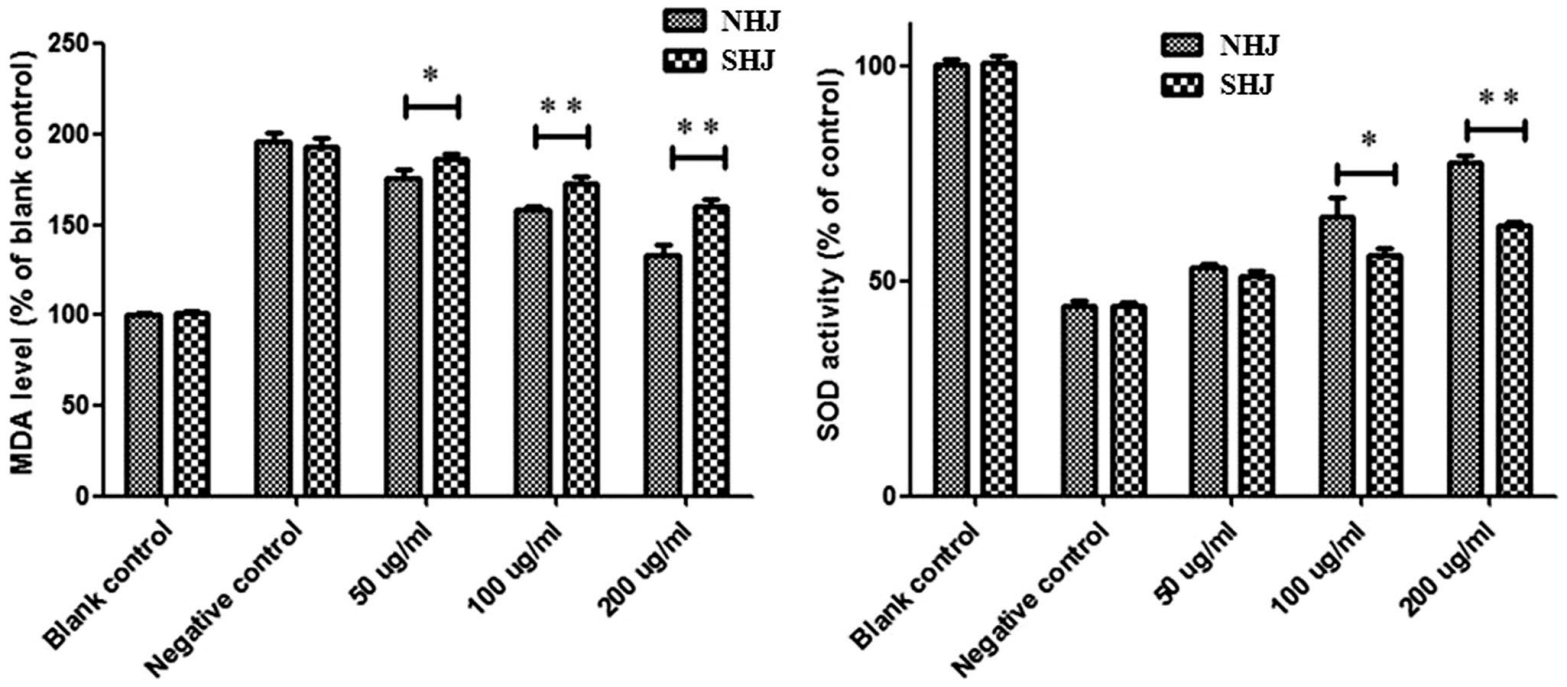
NHJ and SHJ decreased MDA levels and elevated SOD activity in ox-LDL-treated HUVECs. The decrease in MDA levels and elevation of SOD activity was more robust in HUVECs treated with than in HUVECs treated with SHJ. *p < 0.05, **p < 0.01 indicate a significant difference compared to the SHJ group.

MDA is formed during oxidative degeneration and is considered an indicator of lipid peroxidation. SOD is an important free radical scavenger that acts via multiple mechanisms to prevent damage from reactive O_2_^–^. The results showed that compared to the BC group, the ox-LDL group exhibited a dramatic increase in MDA levels and a decrease in intracellular SOD enzyme activity; this increase in MDA levels was reversed by treatment with both NHJ and SHJ, with the effect increasing as the NHJ or SHJ dose increased. These results also showed that the decrease in MDA levels and the elevation of SOD activity were more efficient in HUVECs treated with NHJ compared to HUVECs treated with SHJ (Figure 3).

### Effects of HJ extracts on HUVEC apoptosis

**Figure 4.**
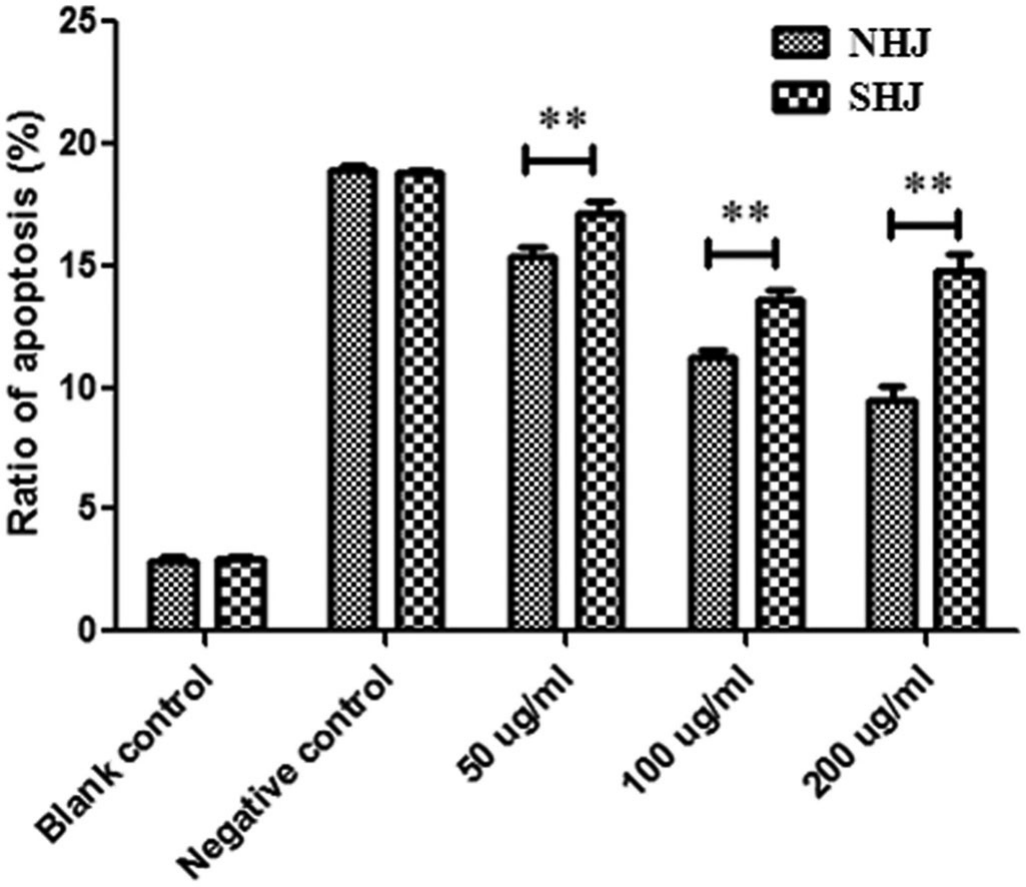
NHJ and SHJ influence apoptosis of ox-LDL-treated HUVECs. NHJ was more effective at inhibiting apoptosis than SHJ at the same dose. **p < 0.01 indicate a significant difference compared to the SHJ group.

Induction of ox-LDL-induced HUVEC apoptosis was evaluated using annexin V-FITC/PI staining. FCM analysis demonstrated that the NC group that was treated only with ox-LDL exhibited more apoptotic cells than the BC group. The results also showed that compared to control treatment, incubation with NHJ significantly reduced the number of apoptotic cells, with the effect increasing as the NHJ increased. However, the number of apoptotic cells only decreased after pre-treatment with 50 or 100 μg/mL SHJ. The number of apoptotic cells was increased in the 200 μg/mL SHJ-treated group compared to 100 μg/mL SHJ-treated group (Figure 4).

## Discussion

*Chrysanthemi Flos* (Ju Hua in Chinese) is a medicinal and edible cognate that is botanically derived from the dry capitulum of *C. morifolium* Ramat. (Xie et al. 2012). In recent years, the importance of *C. morifolium* as a source of potentially novel bioactive compounds has been gradually recognized due to its potential antioxidative effect and its ability to lower blood pressure and blood lipid levels (Sudha et al. 2016). Our previous studies indicated that some chemical transformations, such as hydrolysis of flavonoid glycoside and a significant loss of caffeoylquinic acids, occurred in various cultivars of *C. morifolium* during SF processing (Chen et al. 2015). Moreover, the destruction of hydroxycinnamoylquinic acids in SHJ has a tremendous impact on its antioxidant capacity (Wang S et al. 2014). Nevertheless, the hazardous effects of SHJ have not been adequately studied, and thus, the role of SF in the bioactivities of *C. morifolium* has is not fully understood. In this study, the effects of NHJ and SHJ on oxidative stress-induced antioxidation were compared both *in vivo* and *in vitro*.

Hyperlipidaemia plays critical roles in the pathogenesis of many cardiocerebrovascular diseases by increasing the levels of TC, TG and LDL-C and decreasing the level of HDL-C in the blood. Thus, the lipid regulatory and antioxidative effects HJ extracts on hyperlipidemic rats were first tested. Before the study, a preliminary experiment was carried out in 60 mice, and the data revealed that none of the mice experienced adverse reactions at a dose of 15 (NHJ) or 10 g/kg (SHJ) (data were not shown). While the maximum tolerated doses of NHJ and SHJ are unknown, a dose below 10 g/kg for both NHJ and SHJ is considered safe. Thus, three different doses under 10 g/kg were used in this study. According to Chinese Pharmacopoeia (Chinese Pharmacopoeia Commission 2015), the effective dose of HJ is 2 g/kg, as determined by converting the human equivalent dose to the animal dose. NHJ and SHJ were used at concentrations of 1, 2 and 4 g/kg in the animal experiments. Consistent with previous findings (Wang et al. 2017), the results showed that both NHJ and SHJ significantly decreased serum TG, TC, LDL-C, LDL/HDL and MDA levels and increased serum HDL-C and SOD, with the effect increasing as the NHJ or SHJ dose increased. The results also demonstrated that the lipid regulatory and antioxidant effects of NHJ were significantly more robust than those of SHJ, as expected. There is emerging evidence that sulphur compounds stimulate immune cells and induce the inflammatory (Jacobi-Gresser et al. 2015), and immune cells have reported to be able to affect lipid metabolism (Schaftenaar et al. 2016). It is speculated that residual sulphur compounds in SHJ may be one of the potential reasons in LDL in NHJ and SHJ treatment groups.

After the lipid regulatory and antioxidative effects HJ extracts on hyperlipidemic rats were identified, *in vitro* examination further confirmed these findings in HUVECs. Ox-LDL is associated with atherosclerosis development and is widely used to establish the cellular model of atherosclerosis by exposing to HUVECs (Hu et al. 2019). The different effects of two HJ extracts on the prevention of oxidative damage and dysfunction of HUVECs caused by ox-LDL were explored *in vitro*. Before these tests were carried out, the equilibrium solubility of HJ extracts in the HUVEC culture medium was detected, and the results showed that NHJ was more soluble than SHJ. The sensitivity of HUVECs to HJ extracts was also tested, and the IC_50_ values of NHJ and SHJ were 186.7 and 19.9 mg/mL, respectively. As such, compared to SHJ, NHJ had very little inhibitory effect on HUVEC growth. Therefore, the test doses of 50, 100 and 200 μg/mL were used to further study the antioxidant activity of HJ extracts. The effects of HJ extracts on intracellular ROS, MDA and SOD levels in HUVECs were tested, and the results indicated that the decrease in ROS and MDA levels and elevation of SOD activity was more robust in HUVECs treated with NHJ than those treated with SHJ. Another *in vitro* study of antioxidation showed that extracts from the stems and leaves of *C. morifolium* have very good scavenging effects against DPPH radicals (Kim 2014). Moreover, other cell experiments have also indicated that aqueous *C. morifolium* extracts can relieve damage to pheochromocytoma (PC) 12 cells caused by H_2_O_2_ (Zheng et al. 2015). Some studies have shown that *Chrysanthemum boreale* might be a source of antioxidant flavonoids, especially rutin and isoquercitrin (Kim 2014).

Oxidative stress is caused by an imbalance between the production of various ROS and antioxidant defence, and ROS have been identified as signalling molecules in various pathways, regulating both cell survival and cell death (Wang J et al. 2014). In this study, the percentage of apoptotic cells was increased in the ox-LDL treatment group compared to the control group. Compared to ox-LDL treatment, NHJ significantly reduced the number of apoptotic cells, with the effect increasing as the NHJ dose increased. Several studies have shown that a hot water extract of the flowers of *C. morifolium* effectively inhibits the ox-LDL-induced expression of ICAM-1 and E-selection in HUVECs (Lii et al. 2010). However, in the SHJ-pre-treated groups, the percentage of apoptotic cells decreased at doses of 50 and 100 μg/mL but increased at a dose of 200 μg/mL. It is well known that SHJ can generate sulphur dioxide, which can remain in the treated herbs and alter their original components (Duan et al. 2016). This might explain why the apoptotic cell population increased in the group pre-treated with 200 μg/mL SHJ.

MDA is the aldehyde byproduct of ROS and indirectly reflects the degree of oxidative damage to the liver and endothelial cells. SOD is the only enzyme that employs oxygen free radicals as substrates in aerobic organisms. Thus, it is the most important enzyme in the body and can catalyse the disproportionation reaction of O_2_^–^ to H_2_O_2_, which has a cytoprotective effect by blocking the production of toxic hydroxyl radicals. It has been reported that MDA increases SOD activity simultaneously decreases when lipid peroxidation is enhanced by serum lipid elevation (Niki 2010; Wang et al. 2017). Our results showed that SHJ decreased the activity of serum and cellular SOD and increased the contents of MDA and ROS, thereby enhancing lipid peroxidation, oxidative damage and membrane damage, which is unfavourable for the protection of metabolic functions in the body. This finding indicates that SF has a tremendous impact on the bioactivity and efficacy of HJ.

In our previous research, we found that flavonoid glycoside is transformed into flavonoid aglycone through hydrolysis during SF processing. Moreover, hydroxycinnamoylquinic acids are broken down after SF (Wang S et al. 2014). However, the link between the changes in the compounds and the changes in the antioxidant activity of *Chrysanthemum* is still not clear. In future studies, these different components will be isolated, and their antioxidant activity will be further studied.

## Conclusions

The effects of two HJ extracts on oxidative stress in a hyperlipidemic rat model and on ox-LDL-treated HUVEC apoptosis were comprehensively investigated. Based on the above results, NHJ has better antioxidant potential than SHJ. In future studies, the differences in the chemical composition between the two HJ extracts and their mechanisms in regulating antioxidant and apoptosis will be further clarified.
